# Genome-Wide Approach Identifies Natural Large-Fragment Deletion in ASFV Strains Circulating in Italy During 2023

**DOI:** 10.3390/pathogens14010051

**Published:** 2025-01-10

**Authors:** Claudia Torresi, Roberta Biccheri, Cesare Cammà, Carmina Gallardo, Maurilia Marcacci, Simona Zoppi, Barbara Secondini, Caterina Riverso, Alejandro Soler, Cristina Casciari, Michela Pela, Elisabetta Rossi, Claudia Pellegrini, Carmen Iscaro, Francesco Feliziani, Monica Giammarioli

**Affiliations:** 1 National Reference Laboratory (NRL) for Swine Fever, Istituto Zooprofilattico Sperimentale dell’ Umbria e delle Marche “Togo Rosati”, 06126 Perugia, Italy; r.biccheri@izsum.it (R.B.); c.casciari@izsum.it (C.C.); m.pela@izsum.it (M.P.); e.rossi@izsum.it (E.R.); c.pellegrini@izsum.it (C.P.); c.iscaro@izsum.it (C.I.); f.feliziani@izsum.it (F.F.); m.giammarioli@izsum.it (M.G.); 2 National Reference Center for Whole Genome Sequencing of Microbial Pathogens, Database and Bioinformatic Analysis (GENPAT), Istituto Zooprofilattico Sperimentale dell’Abruzzo e del Molise “G. Caporale”, 64100 Teramo, Italy; c.camma@izs.it (C.C.); m.marcacci@izs.it (M.M.); b.secondini@izs.it (B.S.); 3 European Union Reference Laboratory for African swine fever (EURL), Centro de Investigación en Sanidad Animal (CISA), Instituto Nacional de Investigación y Tecnología Agraria y Alimentaria (INIA), Consejo Superior de Investigaciones Científicas (CSIC), Valdeolmos, 28130 Madrid, Spain; gallardo@inia.csic.es (C.G.); soler.alejandro@inia.csic.es (A.S.); 4 Istituto Zooprofilattico Sperimentale Piemonte, Liguria e Valle d’Aosta “I. Altara”, 10154 Turin, Italy; simona.zoppi@izsto.it; 5 Istituto Zooprofilattico Sperimentale del Mezzogiorno, Portici, 80055 Napoli, Italy; caterina.riverso@izsmportici.it

**Keywords:** African swine fever virus, genotype II, natural deleted strains, large-fragment deletion, experimental infection

## Abstract

African swine fever (ASF), characterized by high mortality rates in infected animals, remains a significant global veterinary and economic concern, due to the widespread distribution of ASF virus (ASFV) genotype II across five continents. In this study, ASFV strains collected in Italy during 2022–2023 from two geographical clusters, North-West (Alessandria) and Calabria, were fully sequenced. In addition, an in vivo experiment in pigs was performed. Complete genomic sequencing of 30 strains revealed large-fragment deletions and translocations. In Alessandria, five samples showed two different deletions in the 5′ genomic region: a ~4340 bp deletion (positions ~9020–13,356 in Georgia 2007/1) in four samples and a 2162 bp deletion (positions 17,837–19,998) in one sample. Another strain showed a truncation of 1950 bp at the 3′ end. In Calabria, strains showed a 5137 bp deletion (positions 10,755–15,891) and ~2 kb truncations in the 3′ region. Two strains showed a translocation from the region 1–2244 to positions 188,631–190,584. In vivo characterization of the deleted strain 22489.4_2312/RC/2023 revealed identical disease progression to the wild-type strain, with severe ASF symptoms in inoculated pigs. This study is the first to report natural deleted strains of ASFV in Italy, revealing unique genomic deletions distinct from those in previously known strains.

## 1. Introduction

The African swine fever (ASF) is a hemorrhagic disease of significant veterinary importance for the pig swine population [1,2,3,4]. In domestic pigs, acute infections can result in a mortality rate of up to 100%, and there are no competent vaccine or treatment options helpful for this disease [4,5]. It is caused by a double-stranded deoxyribonucleic acid (DNA), namely ASF virus (ASFV) [1]. ASFV was assigned to the genus *Asfivirus* and, together with abalone asfa-like virus (AbALV), is the only members of the *Asfarviridae* family to date [1,6,7]. The genome size of ASFV ranges from 170 to 193 kilobase pairs (kbp) [8]; it is composed of a conserved central region (CCR) of about 125 kbp, and two variable ends containing inverted terminal repeats (ITRs) and hairpin loops [8]. The CCR is mainly conserved, but many variations may be accumulated in the 5′ and 3′ ends, the left and right variable regions [9], which are about 40 kbp and 15 kbp long, respectively. With few exceptions, large deletions have generally been found inside the variable terminal regions [10].

Most genes, necessary for virus replication, are located in the center of the genome. The left and the right variable regions of the genome comprise copies of several multigene families (MGFs) including MGF 360, MGF 505/530, MGF 110, MGF 300, and MGF 100 [11]. The gain and loss in copy numbers of these genes affects the length of the genome [11]. MGF 360 is the widest, with 11 to 19 copies, followed by MGF 505/530, with eight to 10 copies, then MGF 110, with five to 13 copies, MGF 300, with three to four copies, and finally MGF 100, with two to three copies [12]. Although the function of the MGF genes is mostly not known [12], they are implicated in translation and transcription, virulence, antigenicity, and immune escape of the ASFV [13].

In general, in ASFV, variations occur predominantly in two regions located close to the 5′ and 3′ ends, which include the ITRs and unique sequences [14]. Differences in the numbers of short tandem repeat sequences (TRS) within the coding and intergenic regions of the genome also contribute to variations [8]. The similarity between family members may vary from less than 30% to more than 90%, indicating that gene duplication within the multigene families is an evolution process. The ASFV genome presents between 151 and 167 predicted open reading frames (ORFs) encoding 54 structural proteins [8]. The encoded genes are closely distributed across both DNA strands [8]. The isolation of virus variants containing deletions in the termini supports that the DNA segments involved are not essential.

The large-scale genome sequencing for ASFV was possible thanks to the development of different methods and platforms for next-generation sequencing (NGS), allowing the identification of genetic differences as single nucleotide polymorphisms (SNPs), insertions and deletions (indels) and rearrangements [15]. Amplicon-based whole-genome sequencing (WGS) involves the use of multiple pairs of overlapping primers to amplify the ASFV viral genome prior to sequencing [16]. However, primer mismatches with the targeted sequences can lead to amplicon failure, which may occur due to recombination events or large deletions within the ASFV genome [16]. Alternatively, the hybrid capture-based method utilizes pre-designed biotinylated RNA baits for the specific enrichment of the ASFV genome. Despite its potential, this approach may be constrained by the high cost of the RNA baits [17].

ASFV WGS indicates that there are different ASFV-B646L-vp72 genotype II natural circulating strains, several of which are distinguished by unique genomic deletions.

One of the most well-known deleted ASF strains is Estonia 2014, identified from wild boar in North-East Estonia in 2014 [18]. Thirteen of the 26 missing genes identified by molecular characterization are associated with MGF110 (1 L–14 L), while three are associated with MGF360 (1 L–3 L). The genes MGF100-1R, L83L, L60L, and KP177R have also been deleted. It is still unknown what process led to this significant reorganization of the genome. Inverse binding of approximately 7 kb from the 3′ to the 5′ end, as well as the deletion of the 5′ end, can be explained by a false separation of head-to-tail concatamers during viral DNA replication [19].

During 2020, Nigeria reported many ASF outbreaks, and the molecular characterization revealed the presence of one isolate, the RV502, characterized by a deletion of 6535 bp (positions 11,760–18,295), and an apparent reverse complement duplication of the 5′ end to the genome at the 3′ end. Nigeria-RV502 mutations do not involve an attenuated virulence in domestic swine [20].

The strains from Ghana present a deletion of eight members of three different MGFs. The comparison between the Ghanaian isolates identified a series of SNPs, some of which led to changes in amino acids and frame shifts affecting genes involved in endosomal trafficking and uncoating, transcription, and immune modulation of the cGAS-STING pathway [21].

Genome sequencing of field variants of ASFV recently isolated in China revealed mutations, deletions, insertions, or short fragment replacements across every isolate in comparison to Pig/HLJ/2018 (HLJ/18). Eleven isolates had a non-hemorrhagic phenotype and spontaneous mutations or deletions in the EP402R gene, similar to the attenuated European strain Lv177WB/Rie1 [22]. Two isolates were found to be extremely lethal when tested in vivo, whereas two other strains had reduced virulence but were highly transmissible [23]. In 2022, Sun and collaborators [10] reported the molecular description of the isolate YNFN202103, characterized by the deletion of three large-fragments (2114, 7772 and 1477 bp in length): the first two in the left variable region and the third in the CCR. The complexity of the ASFV strains currently circulating in China poses a real problem both for the correct diagnosis of the disease and for its epidemiological investigation [24]. A schematic representation of the ASFV natural deleted strains mentioned above is shown in Figure S1.

Moreover, genomic sequencing of African isolates from countries historically infected with ASFV genotype II and recently invaded countries has revealed the circulation of strains with a minor deletion (~550 bp in the MGF 110-4L gene), detected in Madagascar (MAD/01/1998), Mauritius (MAU/01/2007), and Tanzania (TAN/01/2011) [25].

Deletions have also been identified in ASFV genotype I field strains, NH/P68 and OURT88/3, where the EP153R and EP402R genes are affected. These genes are crucial for ASFV virulence, and deletions in them are associated with an attenuated strain phenotype [24].

ASFV was first introduced in the North-west of Italy in January 2022 [26]. ASFV was isolated from wild boar cases and typed as an ASFV-B646L-vp72 genotype II [27]. Later, cases and/or outbreaks were notified in the Central and the Southern peninsula [28] and more than 2543 events were notified to 20 November 2024. After January 2022, only ASFV genotype II has been identified in Italy and all isolates have a high degree of identity with the strains described in other European countries.

The current investigation consists of a component of an extensive project aimed at sequencing the complete genome of ASFV strains circulating in Italy (more than 130 isolates). Among the results of this research has been the identification of 30 strains exhibiting substantial deletions in the 5′ and or 3′ segment of the viral genome. This study presents the molecular characterization of these ASFV variants Moreover, the study describes the clinical characterization performed by in vivo infection to verify the infectivity of the natural deleted strains here described. This was necessary because the clinical manifestation of natural deleted strains is not predictable a priori: they can either have attenuated clinical manifestations, as in the case of Estonia 2014 [18], or maintain a wild-type phenotype, as proposed for Nigeria-RV502 [20].

## 2. Materials and Methods

### 2.1. Specimens

Samples have been collected by the National Reference Laboratory for Swine Fevers (CEREP, IZSUM), according to national surveillance/eradication plan or for confirmation of suspicious cases from affected Italian areas.

Suspensions composed of 10% *w*/*v* spleens or bone marrows were centrifuged for 10 minutes at 1200× *g*. Following the manufacturer’s instructions, viral DNA was extracted using a High Pure PCR Template Preparation Kit (Roche Diagnostics GmbH, Roche Applied Science, Mannheim, Germany). Porcine beta-actin [29] and ASFV were detected in the samples using real-time PCR [30,31].

ASFV-positive samples with ASFV-Ct ≤ 28 were considered suitable for WGS and therefore processed according to the protocol reported in Section 2.2. This report describes the comprehensive characterization of 30 ASFV isolates that, subsequent to WGS, were found to demonstrate significant genomic deletions. In particular, the history of ASFV positive samples classified as ASFV variants is reported in Table 1: six ASFV-positive wild boar samples collected in the province of Alessandria (North-western Italy) between January and February 2023 and 24 domestic pig and wild boar samples in the province of Reggio Calabria (Southern Italy) between May and July 2023.

In accordance with the WOAH Manual of Diagnostic Tests and Vaccines for Terrestrial Animals [31], the viral isolation (Malmquist test) was used to determine whether infectious ASFV was present in all the PCR-positive samples. The presence of live ASFV was verified in the event of hemadsorption (HAD), and the culture supernatant was collected and stored at −80 °C until propagation. The test was repeated by introducing culture supernatants into new, fresh, three-day-old monocyte/macrophage monolayers in the absence of hemadsorption. The lack of live ASFV virus was only verified after three negative results [31]. According to the WOAH Manual, isolates that tested positive for ASFV were titered [31].

### 2.2. Whole Genome Sequencing (WGS) of ASFV-Positive Samples

Viral DNA was extracted using QIAamp UltraSens Virus Kit (Qiagen, Hilden, Germany), following the manufacturer’s instructions. DNA samples were quantified using the Qubit^®^ DNA HS Assay Kit (Thermo Fisher Scientific, Waltham, MA, USA) and subsequently used for library preparation using Illumina^®^ DNA Prep, (M) Tagmentation (96 Samples) (Illumina Inc., San Diego, CA, USA). Using standard 150 bp paired-end reads and the NextSeq 500/550 Mid Output Reagent Cartridge v2 (300 cycle) (Illumina Inc., San Diego, CA, USA), deep sequencing was performed on the NextSeq500. In case of poor results in terms of ASFV specific reads (vertical coverage < 30×), the libraries were enriched using Hybridization Capture for Targeted NGS for ASFV (myBaits, Arbor Biosciences Ann Arbor, MI, USA). Following a quality check and raw read trimming with FastQC v0.11.5 and Trimmomatic v0.36, respectively, the NGS data were analyzed combining a mapping and a de novo analysis, within the GenPat platform of the National Reference Centre for Whole Genome Sequencing of Microbial Pathogens: database and bioinformatic analysis (GENPAT). In detail, the mapping analysis was performed against reference sequence ASFV Georgia 2007/1 (accession n. FR682468.2) by iVar v1.3.1 while the de novo analysis was accomplished using a pipeline called “Filtering and de novo,” which performs mapping of reads against ASFV Georgia 2007/1 reference and de novo assembly of filtered reads by SPAdes 3.13 [32]. De novo contigs were then mapped onto consensus sequences produced by mapping in Geneious Prime^®^ 2021.0.1 in order to assess the presence of deletions, duplications and transpositions in the genome of the analyzed ASFV strains. Genome annotation was performed by GATU software [33], adopting ASFV Georgia 2007/01 as reference, and manually curated with the Ugene software package v. 48.1 [34].

### 2.3. Phylogenetic Analysis

A total of 45 B646L-vp72 full gene sequences were used in the phylogenetic analysis: 30 from the isolates characterized in this study and 15 sequences available on GenBank [35] representing 11 ASFV genotypes. MAFFT was used to perform a multiple alignment of the sequences (http://mafft.cbrc.jp/alignment/software (accessed on 30 July 2024)). The maximum likelihood method (ML) with 1000 bootstrap replications was chosen to infer the evolutionary history, and MEGA 11 was used to show the resulting tree [36].

### 2.4. Animals Experiments

#### 2.4.1. Ethical Statement

The in vivo experiment was performed in Biosafety level 3 (BSL3) facilities at the European Union Reference Laboratory for African swine fever (EURL), Centro de Investigación en Sanidad Animal (CISA), Instituto Nacional de Investigación y Tecnología Agraria y Alimentaria (INIA), Consejo Superior de investigacione scientíficas (CSIC), Madrid, Spain. The in vivo experiments were conducted in accordance with the EC Directive 2010/63/EU and approved by the Animal Experimentation and Laboratory Animal Welfare Committee of CISA-INIA/CSIC and by the Spanish Ethical and Animal Welfare Committee (Ref nº PROEX/090/4.21).

#### 2.4.2. Experimental Design

The experiment involved 12-week-old European hybrid pigs, weighing between 20–25 kg, which were sourced from a commercial farm and allowed a seven-day acclimatization period prior to the commencement of the study. All the animals were tested for ASFV antibodies and ASFV genome using the WOAH indirect immunoperoxidase test (IPT) and the WOAH-real time PCR procedure 2 [31,37] to demonstrate their ASF-free status. The animals had unlimited access to water and were fed pig feed twice a day.

The ASFV has been titered on primary pig monocyte/macrophage cells. Titers of virus were defined as the amount of virus causing HAD or infection, in 50% of infected cell cultures (HAD50/mL).

The title detected was 1 × 10.0^4.25^/20 microliter HAD.

Upon arrival to the BSL3 facilities, the animals have been identified by ear tags. Two pigs (#IP13 and #IP14) were intramuscularly (i.m.) inoculated with 10 hemadsorbing units (HAD) of the Italian 22489.4_2312/RC/2023 HAD ASFV isolate selected. The three remaining pigs (#CC11, #CC12 and #CC15) were housed together with the inoculated pigs as in contact (exposed) pigs.

#### 2.4.3. Clinical Evaluation and Sampling

Daily monitoring of clinical signs was conducted, and a clinical score system was employed to quantify these signs. This system involved summing the values of eight clinical signs recorded each day, as previously detailed [38]. These signs included fever, anorexia, recumbence, skin hemorrhage or cyanosis, joint swelling, respiratory distress, ocular discharge, and gastrointestinal symptoms, with severity assigned on a scale from 0 to 3 (with 3 being the most severe). The total score was recorded as the clinical score (CS), which was also needed to determine humane endpoints. Two times a week, beginning three days after infection (dpi) or three days after exposure (dpe), as well as on the final day of their research period, blood-EDTA, serum, and oral (OS) and nasal (NS) cotton sterile dry swabs (DELTALAB) were collected. At the time of inoculation (day 0), negative control samples were obtained. Eighteen tissue and organ types were collected from each necropsied animal, including liver, spleen, tonsil, heart, lung, kidney, submandibular, retropharyngeal, inguinal, popliteal, mesenteric, mediastinal, gastro-hepatic, splenic and renal lymph nodes, bone marrow, diaphragmatic muscle, and intra-articular tissues of joints. The spleen was analyzed using whole genome sequencing (WGS) according to the protocol detailed in Section 2.2.

#### 2.4.4. Sample Analysis

ASFV detection involved the extraction of DNA from organ homogenates, blood, OS, and NS utilizing the High Pure PCR Template Preparation Kit from Roche Diagnostics GmbH, Roche Applied Science, Mannheim, Germany. Briefly, 10% (*w*/*v*) clarified homogenized tissue suspensions were prepared in PBS. After soaking cotton swabs in 2 mL of PBS, they were vortexed for approximately 15 s, incubated at room temperature for one hour, and then microcentrifuged for 10 min. Before virus identification, supernatants were filtered through 0.45 μm pore size MINISART filters and treated with 0.1% gentamicin sulfate at a concentration of 50 mg/mL for one hour at 4 ± 3 °C. To verify the presence of the ASFV genomic DNA, the real-time PCR [31,39] was performed using undiluted extracted DNA for each sample (Positivity threshold: Ct < 40.0)

Virus isolation (VI) and titration of ASFV: VI was performed on PCR-positive samples using porcine peripheral blood monocytes (PBMs) derived from large white out-bred pigs typically aged between eight and 12 weeks. Preparation of PBMs involved processing un-clotted fresh blood using a mechanical defibrinator following the procedure outlined in the WOAH Manual [31]. After a three-day culture period, PBMs were infected at a 1:10 dilution with the PCR-positive samples. The infected cells were then incubated at 37 °C in a 5% CO_2_ environment for seven days in the presence of red blood cells. Plates were observed over the course of seven days to detect the presence of hemadsorption (HAD). Titration of the ASFVs was performed on PBMs cells to determine the endpoint dilution, and viral titer was assessed as the amount of virus causing HAD in 50% of infected cultures (HAD50/mL) [40].

The commercial ELISA (^®^INGENASA-INGEZIM PPA COMPAC K3 Global Standard Diagnostic, GSD, Madrid, Spain) and the WOAH-IPT [31,37] protocols were performed to detect ASFV-specific antibodies.

## 3. Results

### 3.1. Viral Isolation, Whole Genome Sequencing and Phylogenetic Analysis

Viral isolation was conducted on all ASFV PCR positive samples, but only in some cases have we obtained positive results. In particular, for the samples from the North-West cluster it was not possible to obtain viral isolates. This was probably due to the poor quality of the clinical samples that, in those cases, were collected from dead and decomposing animals. On the contrary, the majority of the samples collected in the Calabria cluster showed the characteristic ASF HAD phenomenon. Among these, the sample isolate 22489.4_2312/RC/2023 was selected to carry out the biological test in order to limit the use of live animals.

Complete genome sequences were obtained for 30 ASFV isolates and submitted to the GenBank database with the accession numbers reported in Table 2. The mean vertical coverage was 207× (min.= 14×, max. = 2174×; Table S1). The sequenced ASFV genomes were 183,365–188,639 bp long with CG contents ranging from 38.42% to 38.56%. The mean genetic diversity among new sequences was low (1.3%), suggesting a certain stability.

Phylogenetic analysis of the full B646L-vp72 gene confirmed that all the Italian strains belong to ASFV genotype II (Figure S2), consistent with the phylogenetic analysis of the partial B646L-vp72 sequence conducted on samples collected from all Italian cases and outbreaks since the first ASFV outbreak in January 2022 [28].

#### 3.1.1. ASFV Italian Natural Deleted Strain Circulating in North-West Cluster

**Figure 1:**
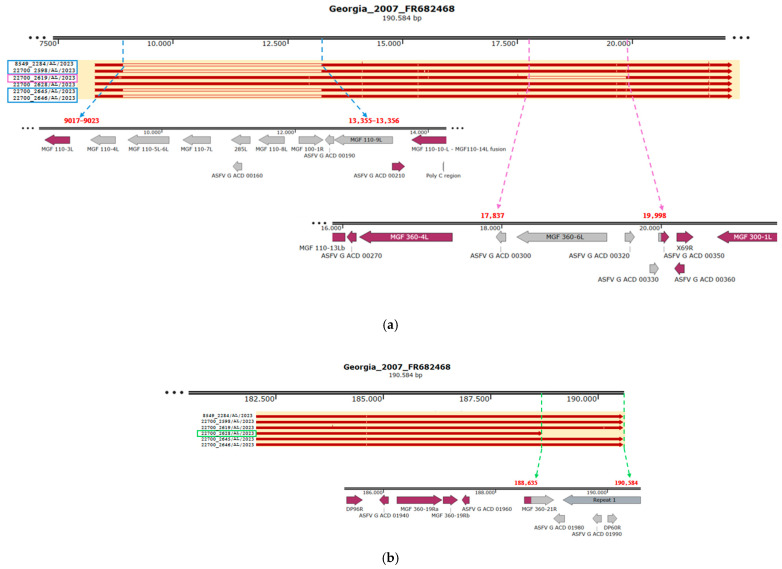
North-West ASF strains that showed two types of large genomic deletions in the 5′ region (**a**). The first, of approximately 4340 bp, affected the region ~9020–13,358 and was identified in four samples (samples marked in blue). The second, spanning 2162 bp in the portion 17,837–19,998, was found in one sample (sample marked in violet). (**b**) The third deletion in the 3′ region, spanning 1950bp in the portion 188,635–190,584, was found in one sample (sample marked in green).

Six ASFV-positive wild boar collected in the province of Alessandria from January to February 2023 exhibited three distinct types of large deletions. In five samples, the 5′ region was affected, resulting in two different deletions (Figure 1a, Table 2). The first deletion, approximately 4340 bp in size, encompassed the region from 9020 to 13,358 of the reference genome ASFV Georgia 2007/1 and was identified in four samples: 8549_2284/AL/2023, 22700_2598/AL/2023, 22700_2645/AL/2023, and 22700_2646/AL/2023. The second deletion, spanning 2162 bp within the region from 17,837 to 19,998 of ASFV Georgia 2007/1, was found in the remaining sample, 22700_2619/AL/2023. Specifically, the first deletion impacted nine genes (MGF 110-4L, MGF 110-5L-6L, MGF 110-7L, 285L, ASFV G ACD 00160, MGF 110-8L, MGF 100-1R, ASFV G ACD 00190, MGF 110-9L), while the second deletion affected five genes (ASFV G ACD 00300, MGF 360-6L, ASFV G ACD 00320, ASFV G ACD 00330, ASFV G ACD 00350). The sequence of ASFV G ACD 00350 is present only in the 3′ portion, with a length of 100 bp compared to 132 bp in Georgia 2007/1.

The remaining sample, 22700_2628/AL/2023, exhibited a 1950 bp deletion in the 3′ region, characterized by a truncation at position 188,365 of ASFV Georgia 2007/1, along with the loss of ASFV G ACD 01980, ASFV G ACD 01990, and DP60R in the ITR, as well as the truncation of MGF 360-21R (Figure 1b, Table 2). In 22700_2628/AL/2023 strain, MGF 360-21R was 657 bp long, compared to 1071 bp in Georgia 2007/1, with the stop codon absent.

#### 3.1.2. ASFV Italian Natural Deleted Strain Circulating in Calabria Cluster

**Figure 2:**
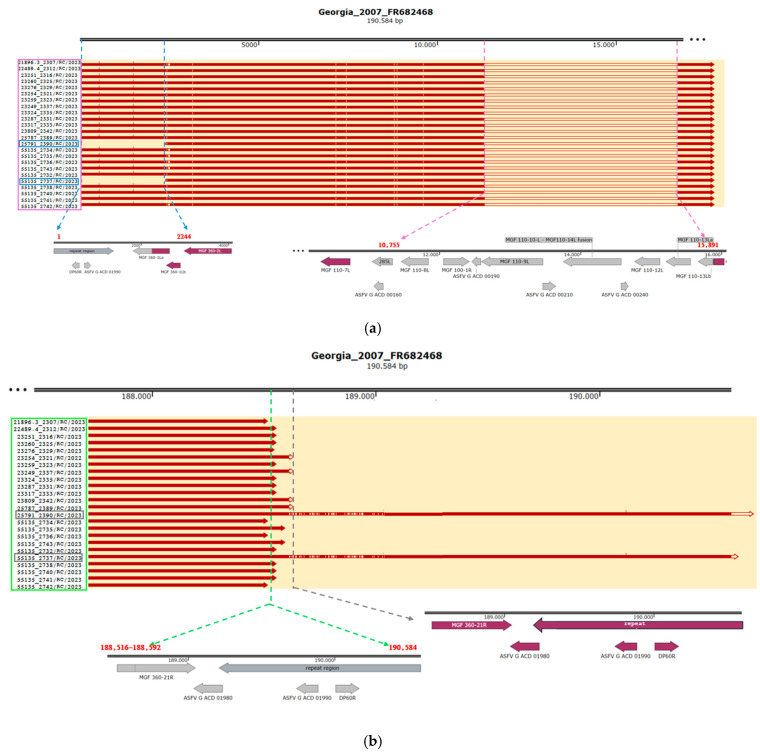
Calabria ASF strains that showed two types of large genomic deletions in the 5’ region (**a**). The first, of approximately 2244bp, affected the region 1−2244 bp and was identified in two samples (samples marked in blue). The second, spanning 5137bp in the portion 10,755–15,891, was found in 22 samples (samples marked in violet). (**b**) The third deletion in the 3′ region was characterized by different lengths (bp) and was found in 24 samples (samples marked in green). A translocation event was found in two samples (samples marked in gray).

**Figure 3:**
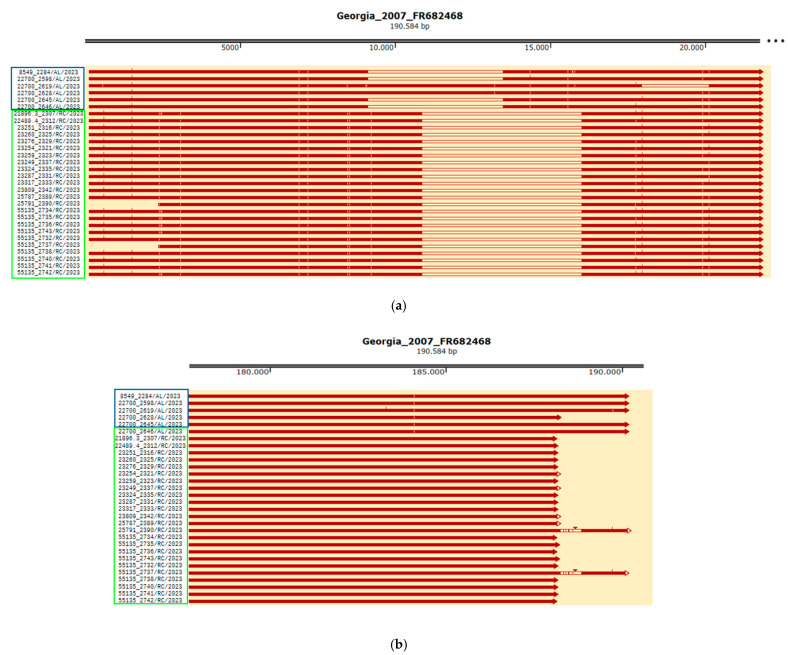
Comparison of North-West and Calabria clusters ASF natural deleted strains. (**a**) 5′ genome region and (**b**) 3′ genome region (Alessandria samples marked in blue and Reggio Calabria samples marked in green).

All samples collected in the Calabria region in 2023 exhibited the same deletion in the 5′ region (Figure 2a, Table 2). This deletion spanned 5137 bp, from positions 10,755 to 15,891 bp in reference to ASFV Georgia 2007/1, and involved 12 genes: 285L, ASFV G ACD 00160, MGF 110-8L, MGF 100-1R, ASFV G ACD 00190, MGF 110-9L, ASFV G ACD 00210, MGF 110-10-L—MGF110-14L fusion, ASFV G ACD 00240, MGF 110-12L, MGF 110-13La, and MGF 110-13Lb. The genomic deletion occurred within MGF 110-13Lb, which was significantly shortened: 126 bp in the deleted strains from Calabria compared to 369 bp in the reference genome Georgia 2007/1 (percent identity: 31%), corresponding to 55 and 122 amino acids, respectively (percent identity: 20%). This deletion partially overlaps with the one observed in naturally deleted strains from the North-west cluster (Figure 3a).

In all samples excepting 25791_2390/RC/2023 and 55135_2737/RC/2023, a second type of deletion affected the 3′ region, causing variable truncated ends approximately situated between locations 188,516 and 188,631 of Georgia 2007/1 (Figure 2b, Table 2). It was 1954–2096 bp long and caused MGF 360-21R to be truncated as well as the deletion of three genes (ASFV G ACD 01980, ASFV G ACD 01990, and DP60R within the ITR). In strains from Calabria, MGF 360-21R was between 537 and 651 bp long, compared to 1071 bp in Georgia 2007/1, with the stop codon absent. This deletion is comparable to that observed in isolate 22700_2628/AL/2023 (Figure 3b).

Two strains, 25791_2390/RC/2023 and 55135_2737/RC/2023, exhibited a 5′ to 3′ translocation (Figure 2a,b, Table 2). Positions 1–2244 of ASFV Georgia 2007/1, absent at the 5′ end, were translocated and inverted at the 3′ end. As a result, the 5′ ITR, along with DP60L and ASFV G ACD 01980 was deleted. At the 5′ end, MGF 360-1La lost 435 bp, resulting in a truncated protein of 133 amino acids, compared to 277 amino acids in Georgia 2007/1 (percent identity: 47.6%). At the 3′ end, the ITR maintained an unchanged structure compared to the reference genome, with ASFV G ACD 01980 missing. The DP60R gene within the ITR displayed an insertion in a homopolymeric sequence (10(A) instead of 9(A) as in Georgia 2007/1), leading to a frameshift and an elongation of the coding sequence by 15 bp. In the strains 25791_2390/RC/2023 and 55135_2737/RC/2023, an 18 bp longer MGF 360-21R was annotated, coding for a 361 amino acid protein with a percent identity of 69.6% compared to MGF 360-21R in Georgia 2007/1. The 5′ to 3′ translocation resulted in an observed elongation of the genomes in the affected strains at the 3′ end, accompanied by an increased presence of the tandem repeat sequence 5′-AGTAATAATTTTAATCTTTAACGCCTACAGCAGT-3′, specifically 12 repeats in 55135_2737/RC/2023 and 14 in 25791_2390/RC/2023, compared to 11 repeats in Georgia 2007/1.

### 3.2. In Vivo Infection

Virus isolation was attempted in 30 PCR-positive clinical organs (kidneys, spleens and bone marrows). The samples collected from the North-West cluster showed negative results after three passages using PBMs, while 19 samples collected in Southern Italy were successfully isolated. Among these, isolate 22489.4_2312/RC/2023 was selected for in vivo characterization at the EURL-CISA-INIA.

**Figure 4:**
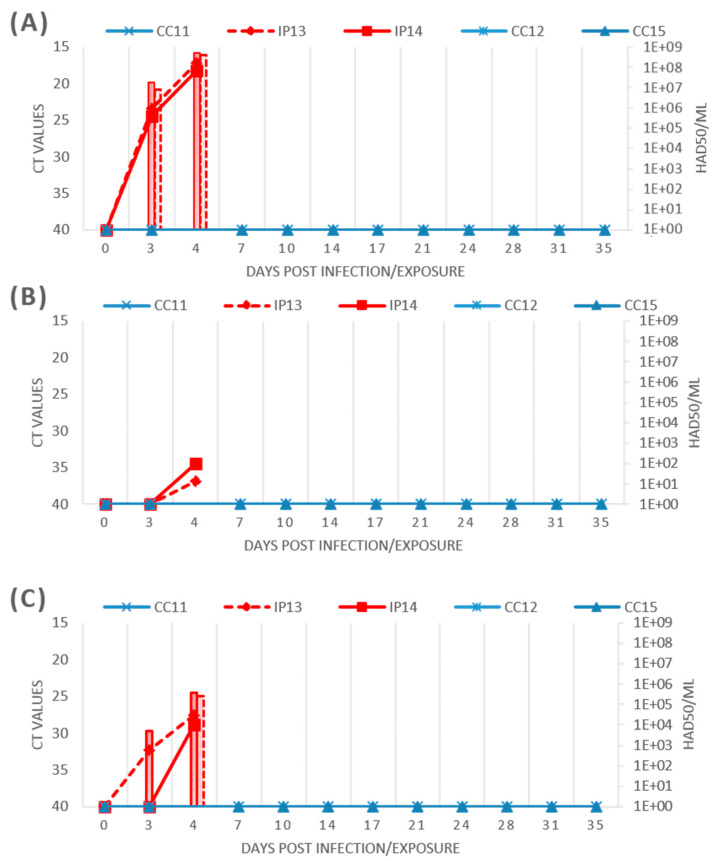
ASFV genome detection by real time PCR (right *X*-axis) overlapped with viral titer (left-*X*-axis) in the blood (**A**), oral swabs (**B**) and nasal swabs (**C**) in domestic pigs infected (IP in red) or exposed (CC in blue) to the hemadsorbing (HAD) Italian genotype II ASFVs.

After an incubation period of three days, the inoculated pigs (IP) exhibited a sudden increase in temperature, exceeding 41 °C by day 4, reaching a clinical score (CS) of nine on that day. Both animals displayed severe anorexia, prostration, and cyanosis in the ears, along with ocular discharge and severe respiratory distress, and were slaughtered for ethical reasons at 4 dpi. The ASFV genome was first detected by PCR at 3 dpi in blood, with a ultimate virus titer of 4.62 × 10^8^ HAD50/mL (8.6 log10) on the day of slaughter (Figure 4A). In oral swabs (OS), ASFV was detected by PCR at 4 dpi, but no virus was isolated (Figure 4B). In nasal swabs (NS), the first positive PCR result appeared on day 3 in Pig IP13, with both pigs shedding virus by day 4, reaching virus titers of 3.10 × 10^5^ HAD50/mL (5.4 log10) (Figure 4C). None of the pigs seroconverted. At post-mortem examination, both IPs presented with skin lesions, including ecchymosis, severe pulmonary edema, and hemorrhagic splenomegaly with an enlarged spleen. Multifocal hemorrhagic lymphadenitis was observed, with the most affected lymph nodes being the gastro-hepatic, renal, and mesenteric. Hemorrhages were also noted in other lymph nodes, such as submandibular, retropharyngeal, and inguinal. Petechial hemorrhages were seen on the kidney surface and upon sectioning, as well as in the mucosa or serosa of the large and small intestines and the epicardium of the heart. The ASFV genome was identified in all the analyzed organs, and the virus was easily isolated in PBMs after one passage, reaching the highest titer in the spleen, with an average titer of 1.75 × 10^8^ HAD50/mL (8.2 log10). WGS was performed on DNA extracted from the spleens of slaughtered animals and the results confirmed the sequence of the strain used for inoculation, 22489.4_2312/RC/2023, with the characteristic 5137 bp deletion in the region 10,755–15,891 of the reference genome and a 2032 bp truncation at the 3′ end.

Exposed pigs were kept in the same enclosure for 35 dpe without showing any clinical signs of illness. Throughout this period, no viremia was detected in their blood, and ASFV was not found in oral or nasal swabs (Figure 4). Additionally, there was no seroconversion, indicating that the pigs did not develop antibodies against ASFV. No virus was detected in any tissues, and no lesions were observed in any of the pigs.

## 4. Discussion

The first introduction of ASFV genotype II into mainland Italy was notified in January 2022 [26], followed by a slow but inexorable spread of the virus across the North-West of the country. Additional introductions of genotype II were documented in Central and Southern Italy during 2022 and 2023. In September 2023, the first ASFV genotype II outbreak was detected on the island of Sardinia, marking the only confirmed escape of ASFV genotype II from the North-Western Italian cluster [41].

Giammarioli and collaborators adopted a multigene approach to genotype Italian strains, leading to the identification of four genetic variants among the isolates collected in Italy during the 2022–2023 epidemic wave [28]. In both the North-West and Calabria clusters, genetic group 3 was identified. Additionally, in the province of Alessandria (North-West cluster), a C/T transition in the O174L gene, codifying for the DNA polymerase X, was detected when compared to Georgia 2007/1. Based on this SNP, four strains (8549_2284/AL/2023, 22700_2598/AL/2023, 22700_2645/AL/2023, and 22700_2646/AL/2023) were clustered into an additional genetic group, namely 26. These four isolates were also affected by the deletion approximately 4300 bp in length reported in this study.

Research indicates that the DNA repair enzymes PolX and LIG in ASFV exhibit low fidelity, contributing to frequent insertions and deletions in its genome [42]. These mutations have driven evolutionary adaptations, often reducing lethality while increasing infectivity. Most mutations appear as large insertions and deletions, particularly affecting multigene families (MGFs) and EP402R.

Studies on ASF genotype II isolates in Germany have described the emergence of a surprising number of variants within a short period, coinciding with frame shifts affecting the O174L gene, speculating on the role of the ASFV DNA PolX gene, oscillating between a repair polymerase and strategic mutagenesis [43].

Despite the ASFV genome’s apparent stability, this study found significant genetic differences between the genotype II ASF viruses from the Calabria and North-West clusters that had never been described before. Within two geographically separate infection clusters in Italy, we found that both domestic pigs and wild boars carried three different forms of 5′ deletions, 3′ truncations, and a 5′-3′ translocation (see A, B, C, D, and E in Table 2). The different types of 5′ deletions overlap ~2600 bp within the coding segment of genes 285L, ASFV G ACD 00160, MGF 110-8L, MGF 100-1R, ASFV G ACD 00190, MGF 110-9L.

The Italian ASFV natural deleted strains exhibited characteristics consistent with those of previously known natural deletion strains, such as the deletion of significant portions of the genome (ranging from 0.5 to 7.7 kb); the genes involved are mainly members of the MGFs 110 and 360; genes adjacent to the deletions sustain rearrangements such as fusions, extensions, or truncations; displacement or duplication from the 5′ to the 3′ end; the left variable region is the most affected portion; when tested in vivo, experiments may show an attenuated phenotype or unchanged virulence compared to the wild-type virus. These profound mutations, therefore, apparently do not disrupt the virus’s life cycle [44], likely due to a compensatory mechanism among the genes belonging to the MGFs. The sporadic emergence of natural deleted isolates, documented in various regions worldwide, presents unpredictable virulence characteristics and has the capacity to cause subclinical manifestations. These isolates persist in the environment and further disseminate, representing a challenge that must be addressed in the design of diagnostic assays.

Among the 26 genes implicated in the deletions and translocations identified in 30 Italian ASFV strains, only three have been ascribed a known function: 285L could play a role in replication or virulence in swine [45,46], MGF 110-7L is involved in phosphorylation and translation [47], and MGF 110-9L is associated with virulence [45,48]. For 23 genes, 11 of which are members of the MGFs 110 and 360, the biological significance remains to be elucidated [8,45,47,49].

Preliminary results from phylogenetic analysis [28] support the hypothesis of multiple ASFV introductions into Italy. In this context, the deletions observed in the Northern region may be attributable to local viral evolution, as the wild type virus is circulating, while in the Southern region, all strains exhibited the same categories of deletions. In Calabria cluster, two hypotheses can be proposed: either a deleted isolate was introduced, or a wild-type isolate (not recovered or sampled) was affected by deletions shortly after its introduction. Considering the minor differences among the Calabrian strains, with small portions still attributable to Georgia 2007/1, the latter hypothesis may be the more plausible one.

Regarding the clinical symptoms of natural ASFV deletions circulating in Italy, in most cases it was not possible to observe the disease symptoms since the positive animals were found dead. Only in a few cases, moribund animals were observed with clinical symptoms attributable to the acute form of the disease (personal observation), which led to the formulation of a suspicion, confirmed by subsequent laboratory analyses.

The in vivo characterization of isolate 22489.4_2312/RC/2023 highlights its high pathogenicity, comparable to its parental ASFV Georgia strain, despite the natural deletion of genomic regions. Specifically, the strain presents a deletion of 5137 bp near the 5′ end and another deletion of 2032 bp at the 3′ end, both involving members of MGFs. Despite these deletions, the strain caused an acute form of the disease in inoculated pigs, demonstrating severe clinical signs and high viral loads, consistent with the virulence of its parental strains. Notably, the exposed contact pigs remained clinically healthy throughout the 35-day observation period. No signs of viremia, viral shedding (in oral or nasal swabs), or seroconversion were detected. Additionally, post-mortem analysis revealed no lesions or presence of ASFV in any tissues from the contact pigs. These findings suggest that, under the specific experimental conditions, the virus was not transmitted from the infected pigs to their naïve pen-mates.

The lack of horizontal transmission could be partially explained by the limited window for virus excretion. In oral swabs, ASFV was detected by PCR at 4 dpi in inoculated pigs, but no virus was successfully isolated. The absence of prolonged or high-level viral shedding may have reduced the likelihood of exposure for the contact pigs. Additionally, the lack of transmission-enhancing factors, such as hemorrhagic signs or significant environmental contamination, further supports the conclusion that the virus did not spread to naïve pigs under these experimental conditions. The absence of transmission could be attributed to limited virus dissemination combined with specific experimental conditions, such as controlled pen hygiene, reduced interactions between pigs, and the short duration of detectable viral shedding, all of which likely minimized opportunities for exposure.

To comprehend the evolutionary impact that the environment causes on ASFV genotype II strains, more research is required to examine the genetic variability data that was gathered from the North-West and Calabria clusters. It is therefore essential to continue investigating isolates collected in the North-West cluster after February 2023 to ascertain whether they coexist with the non-deleted variants, whether they self-extinguished, or whether they have replaced the wild-type.

The emergence of various gene-deleted isolates reveals the complexity of the ASFV circulating in Italy, and the identification of these isolate brings more challenges to the study of ASFV epidemiology in Italy and ASFV diagnosis. It will therefore be fundamental to continue investigating circulating strains of ASFV to define molecular markers that could be characteristic of the Italian territory, and also to further consider the consequences of large genomic deletions on the virulence of ASFV.

This study produced a total of 30 complete ASFV natural deleted genomes. The data have not only significantly increased the scope of complete genomic information of ASFVs from Europe in the public domain, but are providing novel insights into the ASF viruses associated with the wild boar-habitat cycle [50,51] where it is known to play a role in virus maintenance and transmission.

In conclusion, this study is the first report of natural deleted ASFV genotype II circulating in Italy, in the province of Alessandria belonging to the North-West cluster, and in the province of Reggio Calabria belonging to the Calabria cluster; collected between January and February 2023 for the first, and between May and July 2023 for the latter. The deletions, affecting large genomic fragments, differ from those previously described and, according to the data currently available, appear to be characteristic of the region in which they were identified. Two strains exhibited a translocation of 2244 bp from the 5′ to the 3′ end of the genome, and 23 out of 30 isolates showed a loss of approximately 2 kb at the 3′ end. Additionally, only one of the isolated strains was used for experimental infection to minimize the use of live animals. The gene function of many ASFV genes is still poorly understood, especially those belonging to the MGFs, which are typically most impacted by natural deletion events. However, based on the results of the experimental infection presented in this study, it is reasonable to conclude that the deletions affecting certain ASFV strains circulating in Italy do not affect the viral life cycle, pathogenicity, or the packaging of the viral particle. Further studies are therefore required to clarify the biological significance of the deleted genes.

## Figures and Tables

**Table 1:** History and laboratory results for the ASF deleted variants highlighted in this study.

Isolate	Region	Province	Cluster	Collection Date	Host	Sample Type	Ct Mean
8549_2284/AL/2023	Piedmont	Alessandria	North-west	11 January 2023	wild boar	bone marrow	25.15
22700_2598/AL/2023	Piedmont	Alessandria	North-west	14 January 2023	wild boar	spleen	19.72
22700_2619/AL/2023	Piedmont	Alessandria	North-west	26 January 2023	wild boar	spleen	17.84
22700_2628/AL/2023	Piedmont	Alessandria	North-west	27 January 2023	wild boar	kidney	22.32
22700_2645/AL/2023	Piedmont	Alessandria	North-west	24 February 2023	wild boar	bone marrow	21.62
22700_2646/AL/2023	Piedmont	Alessandria	North-west	24 February 2023	wild boar	bone marrow	26.50
21896.3_2307/RC/2023	Calabria	Reggio Calabria	Calabria	3 May 2023	wild boar	spleen	16.88
22489.4_2312/RC/2023	Calabria	Reggio Calabria	Calabria	9 May 2023	domestic pig	spleen	17.17
23251_2316/RC/2023	Calabria	Reggio Calabria	Calabria	11 May 2023	domestic pig	spleen	16.45
23260_2325/RC/2023	Calabria	Reggio Calabria	Calabria	11 May 2023	domestic pig	spleen	17.07
23276_2329/RC/2023	Calabria	Reggio Calabria	Calabria	11 May 2023	domestic pig	spleen	19.37
23254_2321/RC/2023	Calabria	Reggio Calabria	Calabria	13 May 2023	domestic pig	spleen	18.76
23259_2323/RC/2023	Calabria	Reggio Calabria	Calabria	13 May 2023	domestic pig	spleen	16.69
23249_2337/RC/2023	Calabria	Reggio Calabria	Calabria	14 May 2023	domestic pig	spleen	18.48
23324_2335/RC/2023	Calabria	Reggio Calabria	Calabria	14 May 2023	domestic pig	spleen	17.81
23287_2331/RC/2023	Calabria	Reggio Calabria	Calabria	14 May 2023	domestic pig	spleen	19.32
23317_2333/RC/2023	Calabria	Reggio Calabria	Calabria	14 May 2023	domestic pig	spleen	20.40
23809_2342/RC/2023	Calabria	Reggio Calabria	Calabria	16 May 2023	domestic pig	spleen	18.25
25787_2389/RC/2023	Calabria	Reggio Calabria	Calabria	28 May 2023	wild boar	spleen	23.14
25791_2390/RC/2023	Calabria	Reggio Calabria	Calabria	29 May 2023	wild boar	spleen	17.12
55135_2734/RC/2023	Calabria	Reggio Calabria	Calabria	16 June 2023	domestic pig	spleen	20.05
55135_2735/RC/2023	Calabria	Reggio Calabria	Calabria	18 June 2023	domestic pig	spleen	21.43
55135_2736/RC/2023	Calabria	Reggio Calabria	Calabria	18 June 2023	domestic pig	spleen	22.67
55135_2743/RC/2023	Calabria	Reggio Calabria	Calabria	20 June 2023	domestic pig	spleen	18.34
55135_2732/RC/2023	Calabria	Reggio Calabria	Calabria	23 June 2023	wild boar	bone marrow	23.29
55135_2737/RC/2023	Calabria	Reggio Calabria	Calabria	26 June 2023	wild boar	bone marrow	22.44
55135_2738/RC/2023	Calabria	Reggio Calabria	Calabria	27 June 2023	wild boar	bone marrow	16.58
55135_2740/RC/2023	Calabria	Reggio Calabria	Calabria	28 June 2023	domestic pig	spleen	17.82
55135_2741/RC/2023	Calabria	Reggio Calabria	Calabria	28 June 2023	wild boar	bone marrow	23.46
55135_2742/RC/2023	Calabria	Reggio Calabria	Calabria	17 July 2023	domestic pig	bone marrow	26.02

**Table 2:** Genomic overview of the Italian ASF deleted variants characterized in this study. Georgia 2007/1 is the ASFV-B646L-vp72-genotype II reference genome (acc.no. FR682468.2).

Isolate	Acc. Number	Length	5′ Deletion			3′ Deletion			5′ to 3′ Translocation	
			Position in Georgia 2007/1	Length	Deleted Genes	Position in Georgia 2007/1	Length	Deleted Genes	Position in Georgia 2007/1	Deleted Genes
8549_2284/AL/2023	PP317821	186,259	9018–13,356	4339	A					
22700_2598/AL/2023	PP317789	186,250	9023–13,355	4333	A					
22700_2619/AL/2023	PP317797	188,456	17,837–19,998	2162	B					
22700_2628/AL/2023	PP317802	188,639				188,635–190,584	1950	D		
22700_2645/AL/2023	PP317809	186,251	9017–13,356	4340	A					
22700_2646/AL/2023	PP317810	186,250	9017–13,356	4340	A					
21896.3_2307/RC/2023	PP050517	183,370	10,755–15,891	5137	C	188,516–190,584	2069	D		
22489.4_2312/RC/2023	PP050518	183,403	10,755–15,891	5137	C	188,553–190,584	2032	D		
23251_2316/RC/2023	PP050520	183,408	10,755–15,891	5137	C	188,553–190,584	2032	D		
23260_2325/RC/2023	PP050521	183,408	10,755–15,891	5137	C	188,553–190,584	2032	D		
23276_2329/RC/2023	PP182138	183,394	10,755–15,891	5137	C	188,544–190,584	2041	D		
23254_2321/RC/2023	PP182136	183,497	10,755–15,891	5137	C	188,630–190,584	1955	D		
23259_2323/RC/2023	PP182137	183,403	10,755–15,891	5137	C	188,554–190,584	2031	D		
23249_2337/RC/2023	PP050519	183,485	10,755–15,891	5137	C	188,630–190,584	1955	D		
23324_2335/RC/2023	PP050522	183,399	10,755–15,891	5137	C	188,553–190,584	2032	D		
23287_2331/RC/2023	PP182139	183,405	10,755–15,891	5137	C	188,553–190,584	2032	D		
23317_2333/RC/2023	PP182140	183,400	10,755–15,891	5137	C	188,553–190,584	2032	D		
23809_2342/RC/2023	PP050523	183,481	10,755–15,891	5137	C	188,630–190,584	1955	D		
25787_2389/RC/2023	PP050524	183,480	10,755–15,891	5137	C	188,630–190,584	1955	D		
25791_2390/RC/2023	PP182141	183,580	10,755–15,891	5137	C				1–2244, translocated to 188,631–190,584	E
55135_2734/RC/2023	PQ367235	183,371	10,755–15,891	5137	C	188,516–190,584	2069	D		
55135_2735/RC/2023	PQ367236	183,455	10,755–15,891	5137	C	188,592–190,584	1993	D		
55135_2736/RC/2023	PQ367237	183,373	10,755–15,891	5137	C	188,516–190,584	2069	D		
55135_2743/RC/2023	PQ367238	183,448	10,755–15,891	5137	C	188,592–190,584	1993	D		
55135_2732/RC/2023	PQ367239	183,435	10,755–15,891	5137	C	188,553–190,584	2032	D		
55135_2737/RC/2023	PQ367234	183,513	10,755–15,891	5137	C				1–2244, translocated to 188,631–190,584	E
55135_2738/RC/2023	PQ367240	183,405	10,755–15,891	5137	C	188,553–190,584	2032	D		
55135_2740/RC/2023	PQ367241	183,426	10,755–15,891	5137	C	188,553–190,584	2032	D		
55135_2741/RC/2023	PQ367242	183,397	10,755–15,891	5137	C	188,553–190,584	2032	D		
55135_2742/RC/2023	PQ367243	183,365	10,755–15,891	5137	C	188,516–190,584	2069	D		
Legend										
A	MGF 110-4L, MGF 110-5L-6L, MGF 110-7L, 285L, ASFV G ACD 00160, MGF 110-8L, MGF 100-1R, ASFV G ACD 00190, MGF 110-9L									
B	ASFV G ACD 00300, MGF 360-6L, ASFV G ACD 00320, ASFV G ACD 00330, ASFV G ACD 00350									
C	285L; ASFV G ACD 00160; MGF 110-8L;MGF 100-1R; ASFV G ACD 00190; MGF 110-9L; ASFV G ACD 00210; MGF 110-10-L–MGF110-14L fusion; ASFV G ACD 00240; MGF 110-12L; MGF 110-13La; MGF 110-13Lb partial									
D	MGF 360-21R partial, ASFV G ACD 01980, ASFV G ACD 01990, DP60R (inverted terminal repeat)									
E	5″: inverted terminal repeat, DP60L; ASFV G ACD 01990; MGF 360-1La partial. 3″: ASFV G ACD 01980									

## Data Availability

The original data presented in the study are openly available in National Center for Biotechnology Information (NCBI) at https://www.ncbi.nlm.nih.gov.

## Supplementary Materials

The following supporting information can be downloaded at: https://www.mdpi.com/article/10.3390/pathogens14010051/s1, Figure S1: Schematic representation of natural deleted strains mentioned in the introduction of this study. This description is not intended to be exhaustive of all the available knowledge, as this is not the intent of the study. The accession numbers of the sequences are listed below: Georgia 2007: FR682468.2; Estonia 2014: LS478113.1; Nigeria RV502: OP672342.1; Ghana 34: OP718533.1; Ghana 35: OP479889.1; Ghana 40: OP718534.1; Ghana 62: OP718535.1; China YNFN202103: ON400500.1. Figure S2: Maximum likelihood (ML) phylogenetic tree of 45 ASFV B646L/p72 full gene. The 30 ASFV Italian strains are represented with a black dot. The whole sequence of the B646L-vp72 gene is available for only 11 out of the 23 ASFV genotypes. The accession numbers of the sequences used in the phylogenetic analysis are listed below: E75: NC_044958.1; BA71V: NC_001659.2; Georgia 2007: FR682468.2; Warmbaths: NC_044950.1; Warthog: AY261366.1; Tengani62: AY261364.1; Mkuzi 1979: AY261362.1; NAM P1/1995: PP107957.1; Malawi Lil-20/1 (1983): AY261361.1; R35: MH025920.1; Ken06.Bus: NC_044946.1; Ken05/Tk1: NC_044945.1; Kenya 1950: AY261360.1; TAN/08/Mazimbu: ON409981.1; Pretoriuskop/96/4: AY261363.1. Table S1. The vertical coverage calculated for Italian ASF deleted variants.
