# Supplementary material for: Genome-Wide Approach Identifies Natural Large-Fragment Deletion in ASFV Strains Circulating in Italy During 2023
Source: Pathogens. 2025 Jan 10;14(1):51. doi: 10.3390/pathogens14010051 (PMC11769418; doi:10.3390/pathogens14010051)
Supplement: Supplementary file 1 [file pathogens-14-00051-s001.zip › suppl materials_T1_S1_S2/Table S1.pdf]

Isolate	Vcov
8549_2284/AL/2023	112
22700_2598/AL/2023	41
22700_2619/AL/2023	37
22700_2628/AL/2023	129
22700_2645/AL/2023	69
22700_2646/AL/2023	32
21896.3_2307/RC/2023	608
22489.4_2312/RC/2023	149
23251_2316/RC/2023	133
23260_2325/RC/2023	70
23276_2329/RC/2023	2172
23254_2321/RC/2023	60
23259_2323/RC/2023	119
23249_2337/RC/2023	70
23324_2335/RC/2023	89

Isolate	Vcov
23287_2331/RC/2023	132
23317_2333/RC/2023	14
23809_2342/RC/2023	111
25787_2389/RC/2023	19
25791_2390/RC/2023	22
55135_2734/RC/2023	130
55135_2735/RC/2023	306
55135_2736/RC/2023	116
55135_2743/RC/2023	333
55135_2732/RC/2023	40
55135_2737/RC/2023	70
55135_2738/RC/2023	818
55135_2740/RC/2023	56
55135_2741/RC/2023	76
55135_2742/RC/2023	103
