# Supplementary figures and images for: Genome-Wide Approach Identifies Natural Large-Fragment Deletion in ASFV Strains Circulating in Italy During 2023
Source: Pathogens. 2025 Jan 10;14(1):51. doi: 10.3390/pathogens14010051 (PMC11769418; doi:10.3390/pathogens14010051)

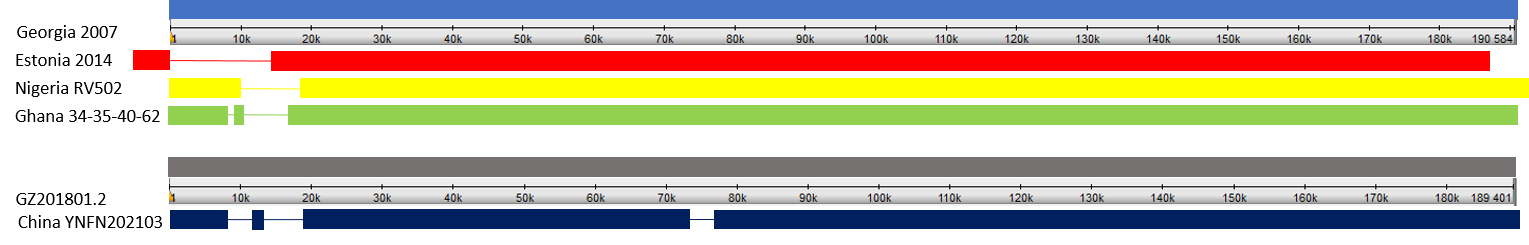

Supplement: Supplementary file 1 [file pathogens-14-00051-s001.zip › suppl materials_T1_S1_S2/Fig S1_scheme.tif]

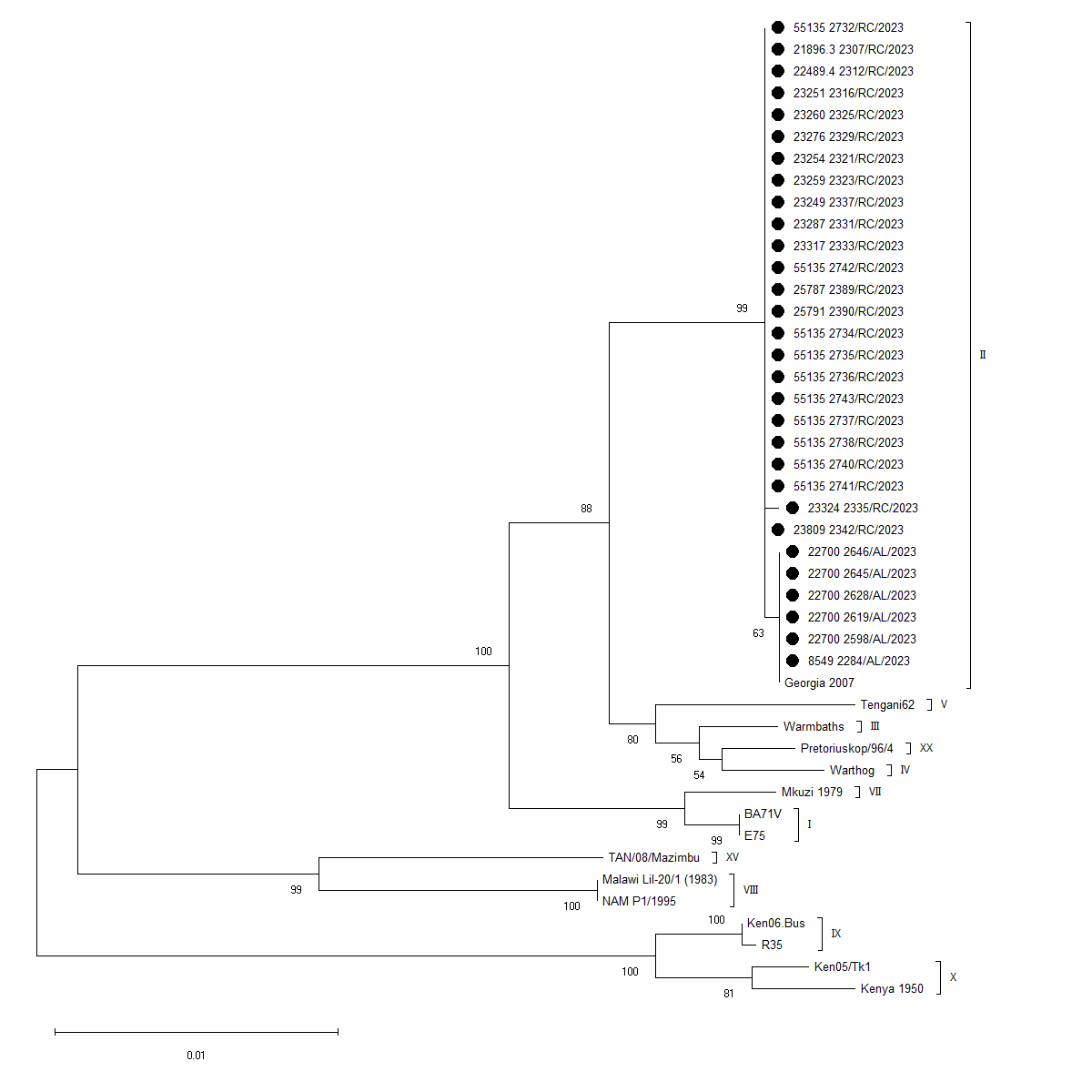

Supplement: Supplementary file 1 [file pathogens-14-00051-s001.zip › suppl materials_T1_S1_S2/Figure S2 VP72 full gene tree.tif]
